# Tinea and Tattoo: A Man Who Developed Tattoo-Associated Tinea Corporis and a Review of Dermatophyte and Systemic Fungal Infections Occurring Within a Tattoo

**DOI:** 10.7759/cureus.21210

**Published:** 2022-01-13

**Authors:** Philip R Cohen, Christopher S Crowley, Christof P Erickson, Antoanella Calame

**Affiliations:** 1 Dermatology, University of California, Davis Medical Center, Sacramento, USA; 2 Dermatology, San Diego Family Dermatology, National City, USA; 3 Dermatology, Compass Dermatopathology, San Diego, USA; 4 Dermatology/Dermatopathology, Compass Dermatopathology, San Diego, USA; 5 Dermatology, Scripps Memorial Hospital, La Jolla, USA

**Keywords:** tinea, tattoo, sporotrichosis, pigment, ink, infection, fungus, dermatophyte, corporis, aspergillus

## Abstract

Fungal infections may occur within tattoos. These include not only dermatophyte infections (tattoo-associated tinea) but also systemic mycoses (tattoo-associated systemic fungal infections). The PubMed search engine, accessing the MEDLINE database, was used to search for all papers with the terms: (1) tinea and tattoo, and (2) systemic fungal infection and tattoo. Tattoo-associated tinea corporis has been observed in 12 individuals with 13 tattoos; this includes the 18-year-old man who developed a dermatophyte infection, restricted to the black ink, less than one-month after tattoo inoculation on his left arm described in this report.

Tattoo-associated tinea typically occurred on an extremity in the black ink. The diagnosis was established either by skin biopsy, fungal culture, and/or potassium hydroxide preparation. The cultured dermatophytes included *Trichophyton rubrum*, *Epidermophyton floccosum*, *Microsporum canis*, *Microsporum gypseum*, and *Trichophyton tonsurans*. Several sources for the tinea were documented: autoinfection (two patients), anthrophilic (tinea capitis from the patient’s son), and zoophilic (either the patient’s cat or dog). Three patients presented with tinea incognito resulting from prior corticosteroid treatment. Tinea appeared either early (within one month or less after inoculation during tattoo healing) in six patients or later (more than two months post-inoculation in a healed tattoo) in six patients.

Injury to the skin from the tattoo needle, or use of non-sterile instruments, or contaminated ink, and/or contact with a human or animal dermatophyte source are possible causes of early tinea infection. Tattoo ink-related phenomenon (presence of nanoparticles, polycyclic aromatic hydrocarbons, and cytokine-enhancement) and/or the creation of an immunocompromised cutaneous district are potential causes of late tinea infection. Treatment with topical and/or oral antifungal agents provided complete resolution of the dermatophyte for all the patients with tattoo-associated tinea.

Tattoo-associated systemic fungal infection has been reported in six patients: five men and one patient whose age, sex, immune status, and some tattoo features (duration, color, and treatment) were not reported. The onset of infection after tattoo inoculation was either within less than one month (two men), three months (two men), or 69 months (one man). The tattoo was dark (either black or blue) and often presented as papules (three men) or nodules (two men) that were either individual or multiple and intact or ulcerated. The lesion was asymptomatic (one man), non-tender (one man), or painful (one man). The systemic fungal organisms included *Acremonium* species, *Aspergillus fumigatus*, *Purpureocillium lilacinum*, *Saksenaea vasiformis*, and *Sporothrix schenckii*. Contaminated tattoo ink was a confirmed cause of the systemic fungal infection in one patient; other postulated sources included non-professional tattoo inoculation, infected tattooing tool and/or ink in an immunosuppression host, and contaminated ritual tattooing instruments and dye. Complete resolution of the tattoo-associated systemic fungal infection occurred following systemic antifungal drug therapy.

In conclusion, several researchers favor that tattoo inoculation can be implicated as a causative factor in the development of tattoo-associated tinea; however, in some of the men, tattoo-associated systemic fungal infection may have merely been coincidental.

## Introduction

Dermatophyte infections can be caused by *Epidermophyton*, *Microsporum*, and *Trichophyton* organisms. They can affect the hair, nails, and skin. Tinea corporis is a dermatophyte infection of the skin [1-8].

Tattoos are a form of body adornment. They can be associated with cutaneous complications such as hypersensitivity reactions to the tattoo ink and skin cancer. In addition, albeit less commonly, either tinea or a systemic fungal infection has developed within a tattoo [8,9].

A man who developed tinea corporis that was located within a black tattoo on his forearm and presented with tinea incognito is described. The purpose of this paper is to review the features of individuals with dermatophyte infection that has been observed to occur within a tattoo. In addition, the rationale for this report is to summarize the characteristics of persons with tattoo-associated systemic fungal infections. These issues are of importance since they provide evidence of a potential role of tattooing as a contributing etiology for dermatophyte and systemic fungal infection within a tattoo [1-15].

## Case presentation

A healthy 18-year-old Caucasian man presented for evaluation of a new asymptomatic red rash that he noticed on his left upper extremity. Four months earlier, a green and red tattoo had been inoculated on his left forearm. Within a month of receiving the tattoo, the skin lesion appeared only on an area of green ink.

He was evaluated by a physician. A bacterial culture was performed, and he was prescribed double-strength trimethoprim-sulfamethoxazole twice daily for ten days and topical 0.1 percent triamcinolone cream twice daily. The culture was negative for bacteria, and he continued to use the corticosteroid cream.

**Figure 1:**
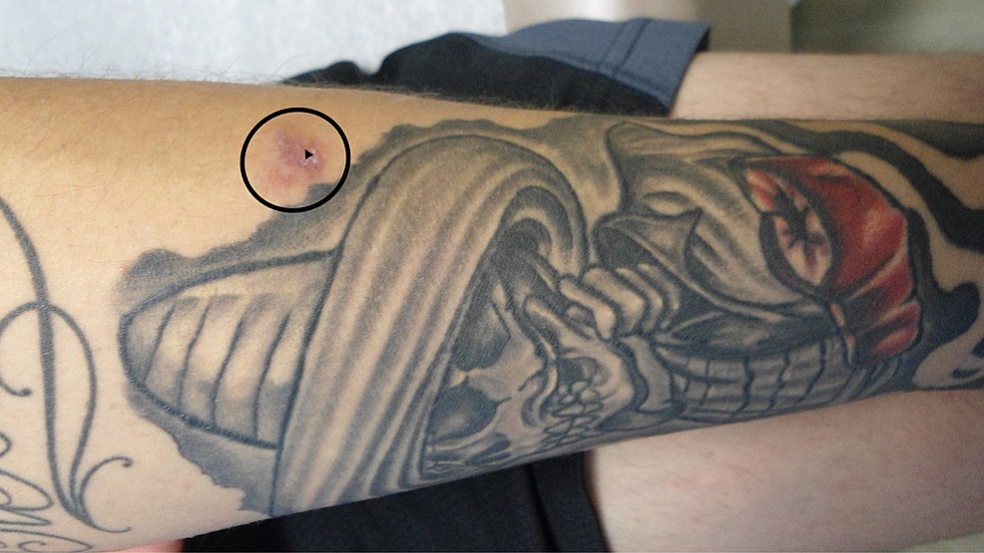
Tinea corporis originating within the green tattoo ink on the proximal left forearm The left forearm of an 18-year-old man shows an asymptomatic erythematous 10 x 10 millimeter scaly plaque that originated within the green tattoo ink and had extended into the adjacent skin (black oval). Four months earlier, a green and red tattoo had been inoculated on his left forearm four months earlier. The skin lesion initially appeared in an area of green ink within a month of receiving the tattoo. He was prescribed an oral antibiotic for ten days and a mid-potency topical corticosteroid cream which was continually applied to the lesion for three months. A four-mm biopsy, using the punch technique was performed; the biopsy site (black triangle) was selected so that the healed scar from the procedure would not be in the tattoo. The chronic topical use of the 0.1 percent triamcinolone cream resulted in an atypical appearance of the dermatophyte infection: tinea incognito.

Three months later, the rash was still present and had enlarged; he was seen by a dermatologist. Cutaneous examination showed a non-tender, 1.0 x 1.0 centimeter erythematous plaque with superficial scaling on his left forearm. The plaque, which had originated in the green tattoo ink, had extended to the adjacent skin (Figure 1).

The dermatologist, attempting to establish a diagnosis of the patient’s new and enlarging cutaneous condition, recommended a biopsy of the skin lesion. Since the patient did not want the skin biopsy to leave a scar on his new tattoo, the four-millimeter biopsy of the plaque was performed using the punch technique at a location that did not include the underlying tattoo. In addition, another bacterial culture was performed; it was negative for bacteria.

**Figure 2:**
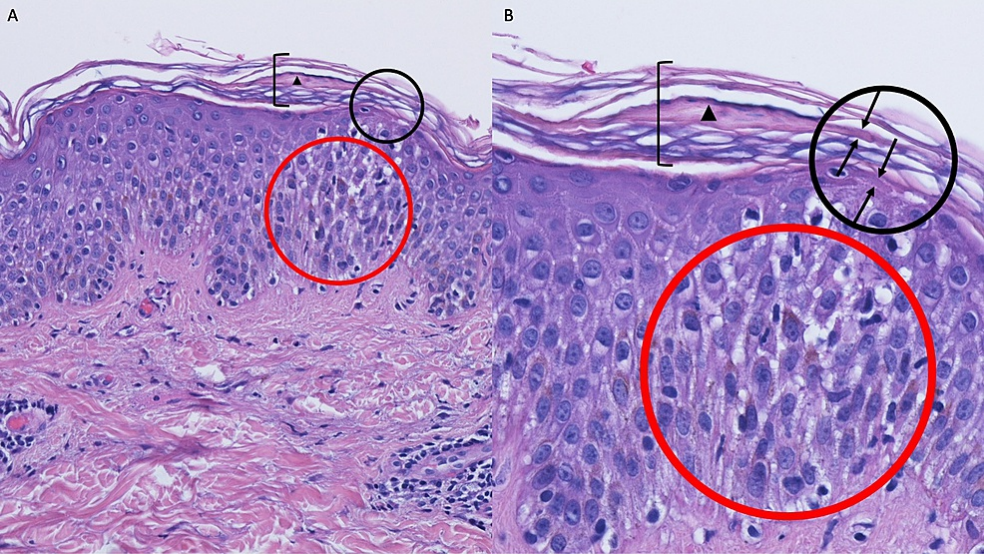
Hematoxylin and eosin-stained sections of a dermatophyte infection within a tattoo which presented as tinea incognito The tissue specimen shows hyperkeratosis (thickening of the stratum corneum) (black bracket) with focal parakeratosis (mounds of retained keratinocyte nuclei within the thickened stratum corneum) (black triangle) overlying the mildly acanthotic (thickened) epidermis. Fungal hyphae (between black arrows in the black oval) are present in the stratum corneum. There is focal spongiosis (widening of the intercellular spaces) (red oval) in the epidermis and a sparse lymphocytic infiltrate, predominantly present around the blood vessels, in the upper dermis (hematoxylin and eosin: A, x10; B. x20).

**Figure 3:**
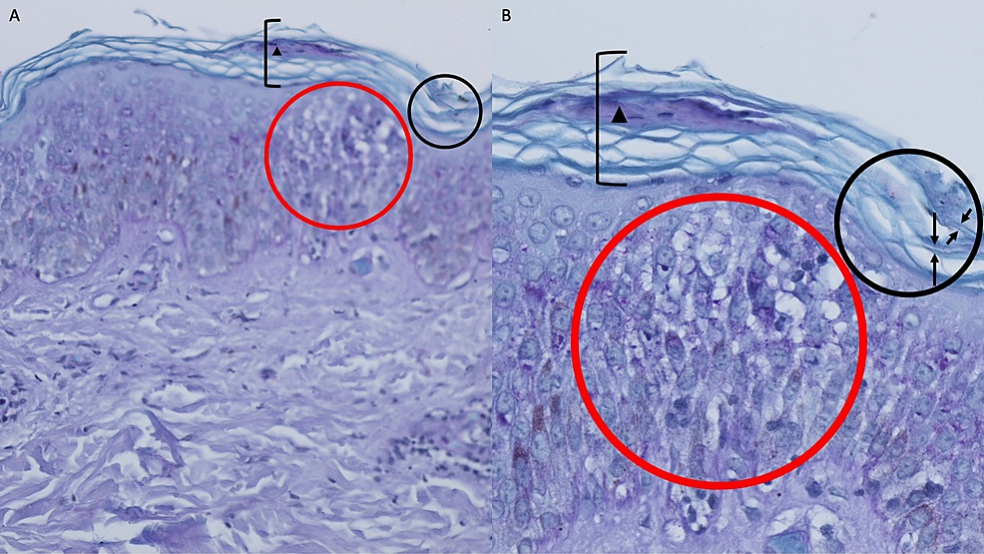
Periodic acid-Schiff (PAS)-stained sections of tattoo-associated tinea Microscopic examination demonstrates focal mounds of parakeratosis (black triangle) within the hyperkeratotic stratum corneum (black bracket). In the stratum corneum, fungal hyphae (between black arrows in the black oval) are present. There is mild acanthosis and focal spongiosis (red oval) of the epidermis. In the upper dermis, there is a sparse perivascular infiltrate of lymphocyte (periodic acid-Schiff: A, x10; B, x20).

Microscopic examination of the tissue specimen showed thickening of the stratum corneum (hyperkeratosis) with focal mounds of retained epithelial cell nuclei within the thickened stratum corneum (parakeratosis) overlying the mildly thickened (acanthotic) epidermis. There were focal areas showing widening of the intercellular spaces (spongiosis) in the epidermis. There was a sparse, predominantly perivascular, infiltrate of lymphocytes in the upper dermis. Both hematoxylin and eosin and periodic acid-Schiff (PAS) stains demonstrated fungal hyphae in the stratum corneum (Figures 2, 3).

Correlation of the clinical presentation and pathologic findings established the diagnosis of tinea corporis originating within a tattoo; the persistent use of the topical corticosteroid cream had resulted in an atypical presentation of his dermatophyte infection: tinea incognito. The 0.1 percent triamcinolone cream was discontinued. He was treated with two percent ketoconazole cream twice daily to the affected area on his forearm.

Follow-up examination, four weeks later, showed complete resolution of his tattoo-associated tinea infection.

## Discussion

An atypical presentation of tinea corporis (tinea incognito) occurred within a recently inoculated tattoo on the forearm of a healthy young man. The infection was initially misinterpreted as a bacterial infection and dermatitis. When the lesion failed to resolve after treatment with oral antibiotics and a mid-potency topical corticosteroid cream, the microscopic evaluation of a skin biopsy from the lesion established the diagnosis of a dermatophyte infection. It is important for clinicians evaluating patients who have tattoos to be aware of the possibility of tinea corporis or a systemic fungal infection originating within the tattoo. This paper discusses tattoo-associated tinea, tattoo-associated systemic fungal infections, and whether the relationship between tattoos and tinea or systemic fungal infections is bona fide or coincidental.

Several infections have been observed to occur within a tattoo [8,9]. These include not only bacterial and mycobacterial infections, but also viral infections [8,16]. In addition, tattoo-associated dermatophyte infections (similar to tinea corporis in the man in this report) and systemic fungal infections have been noted to appear within tattoos [1-15].

The history of published reports of tattoo-associated tinea is summarized in Table 1 [1-7]. The first patients were described by Brancaccio et al in 1981. Trichophyton rubrum was cultured not only from the multicolored (including black) tattoo on the right arm of a 35-year-old man that had been present for 15 years, but also from his tinea pedis; his pruritic dermatophyte lesions developed one week after having the same tattoo restored four weeks earlier. The second patient, a 22-year-old man, had a tattoo of six-years duration on his arm when he was evaluated; his pruritic lesions of tinea, that grew Epidermophyton floccosum in culture, appeared three weeks earlier in the tattoo [1].

**Table 1:** History of tattoo-associated tinea Abbreviations: CR, current report; E, *Epidermophyton*; M, *Microsporum*; T, *Trichophyton*; USA, United States of America

Author	Year	Country	Comment	Reference
Brancaccio et al.	1981	USA	Initial report of dermatophyte (E. floccosum or T. rubrum)-infected tattoos in two men. The researchers coined the term “tinea in tattoo” and suggested “locus minoris resistentiae” as a possible pathogenesis for this phenomenon.	[1]
Ammirati	2004	USA	Initial report of a woman with tinea in tattoo. The same fungal organism (T. tonsurans) was cultured from the tattoo and her five-year-old son’s scalp tinea capitis.	[2]
Ishizaki et al	2012	Japan	Tinea (M. gypseum) in tattoo presenting as tinea incognito and mimicking an allergic reaction to tattoo pigment following cosmetic eyebrow tattooing.	[3]
Oanta and Irimie	2016	Romania	Tinea (M. canis) occurring on both black and green ink of a tattoo. The woman’s pet dog had culture-proven mycosis caused by the same fungal organism.	[4]
Gathings et al.	2018	USA	Tinea incognito (potassium hydroxide preparation-confirmed) in a red tattoo in a man with a chronically treated allergic reaction to tattoo red dye. He had clinical features of tinea pedis and toenail onychomycosis. The researchers postulated that the tattoo dye allergic reaction treatment created an immunocompromised district enabling the tinea in tattoo.	[5]
Panda et al	2019	India	A series of four patients with tinea (T. rubrum in three patients and E. floccosum in one patient) lesions confined to their black tattoos that developed when the tattoos were more than two months old. The researchers postulated a loss of local immunity caused by the black ink since the delayed onset of the tinea infection excluded tattoo instrument-acquired infection.	[6]
Schwob and Kluger	2020	France	Tinea (M. canis) in two separate black tattoos in a woman whose sister (living in the same house) had four similar lesions on her trunk. Her cat had veterinarian-confirmed ringworm that presented as hair loss. The researchers, based on tattoo duration, categorized tinea in tattoo into those associated with either early dermatophyte infection on a recent (healing) tattoo or late tinea infection on an older (and healed) tattoo.	[7]
Cohen et al.	2022	USA	Tinea incognito (skin biopsy-confirmed) in a recently inoculated black tattoo. The researchers also comprehensively reviewed the features of previously reported patients with tattoo-associated tinea and tattoo-associated systemic fungal infections.	CR

It was more than 20 years later before Ammirati reported the next patient with tattoo-associated tinea in 2004. The investigator described a woman who developed pruritic, peripherally expanding concentric lesions which had a vesiculopustular border that developed within the black tattoo that had been inoculated 18 days earlier. *Trichophyton tonsurans* was cultured from both the tattoo-related tinea lesions on her leg and the tinea capitis site on her five-year-old son’s scalp [2].

Tattoo-associated tinea incognito was subsequently described in 2012. A 63-year-old woman developed *Microsporum gypseum*-related dermatophyte skin lesions in the black cosmetic eyebrow tattoos that had been performed four weeks earlier. They were initially mistaken for an allergic reaction to the tattoo pigment, treated topical and systemic corticosteroids for a month, and spread to involve her forehead and cheeks prior to the actual diagnosis of tinea faciei being established [3].

In addition to the man in this report, another patient with tattoo-associated tinea incognito was also reported in 2018. The other patient was a 52-year-old man with evidence of tinea pedis and toenail onychomycosis whose right calf tattoo of four years had received 18 consecutive months of topical treatment (with both a calcineurin inhibitor and a corticosteroid) for an allergic reaction to the red tattoo pigment. Three months earlier, the tattoo reaction had enlarged and became more pruritic; a potassium hydroxide preparation of the current erythematous and scaling patch revealed septate fungal hyphae [5].

In 2016, a 29-year-old woman with tattoo-associated *Microsporum canis* on her left calf was described. The dermatophyte skin lesions appeared within the tattoo 18 days after inoculation. Her pet dog also had *Microsporum canis* mycosis [4].

Four patients were reported by investigators in 2019. All the individuals had a black tattoo of more than two months duration. Either *Trichophyton rubrum* (three patients) or *Epidermophyton floccosum* were cultured [6].

Recently, another woman with black tattoo-associated *Microsporum canis* was described; tinea lesions developed on her right forearm 12 days after inoculation and on her left thigh six days after inoculation. Her sister, who lived with the patient, had similar lesions on her tattoo-free abdomen. In addition, the patient’s pet cat had ringworm-related hair loss that was begin treated by the veterinarian [7].

The epidemiologic features and clinical characteristics of tattoo-associated tinea are summarized in Table 2 and Table 3 [1-7]. Including the man in this report, 12 patients with dermatophyte infection within a tattoo have been described. The individuals have been observed in the United States of America (five patients), India, (four patients), France (one patient), Japan (one patient), and Romania (one patient) (Table 1) [1-7].

**Table 2:** Epidemiologic features of tattoo-associated tinea Abbreviations: A, age (years); Bx, biopsy; C, case; CR, current report; Cult, culture (fungal); E, *Epidermophyton*; KOH, potassium hydroxide preparation; M, *Microsporum*; NS, not stated; Ref, reference; T, *Trichophyton*; Tat dur, Tattoo duration (the number of months from inoculation of the tattoo until the patient became aware of the dermatophyte lesion within the tattoo); W, woman; <, less than; >, more than; +, positive for ^a^Tinea-related lesions appeared on the right forearm tattoo 0.4 months (12 days) after tattoo inoculation and on the left thigh tattoo 0.2 months (six days) after tattoo inoculation.

C	A	Sex	Tat dur	Diagnosis	Source	Ref
1	18	Man	<1	Bx: + hyphae	Unknown	CR
2	22	Man	71.25	KOH: + hyphae; Cult: E. floccosum	NS	[1] C2
3	27	W	0.4, 0.2^a^	KOH: + hyphae; Cult: M. canis	A veterinarian-confirmed that ringworm was the cause of hair loss in the patient’s pet cat and started antifungal treatment; also, the patient’s sister--who lived in the same home--had four lesions (similar to those of the patient) on her tattoo-free abdomen.	[7]
4	29	W	0.6	KOH: + hyphae; Cult: M. canis	The pet dog had culture-confirmed M. canis mycosis.	[4]
5	35	Man	0.25	KOH: + hyphae; Cult: T. rubrum	The patient also had KOH-positive tinea pedis and tinea cruris; the tinea pedis was also culture-positive for T. rubrum.	[1] C1
6	52	Man	45	KOH: + hyphae	The patient also had clinical lesions of tinea pedis (scaling on toe web between fourth and fifth toe) and tinea unguium (yellow, thick, and dystrophic toenails).	[5]
7	63	W	1	KOH: + hyphae; Cult: M. gypseum	The researchers postulated that the pet dog’s paws acquired the geophilic fungal organism during walks; however, the veterinarian did not find any lesions on the dog.	[3]
8	NS	W	0.5	KOH: + hyphae; Cult; T. tonsurans	The patient’s five-year-old son had culture-positive T. tonsurans tinea capitis	[2]
9	NS	NS	>2	Cult: T. rubrum	NS	[6] C1
10	NS	NS	>2	Cult: T. rubrum	NS	[6] C2
11	NS	NS	>2	Cult: T. rubrum	NS	[6] C3
12	NS	NS	>2	Cult: E. floccosum	NS	[6] C4

**Table 3:** Clinical characteristics of tattoo-associated tinea Abbreviations: C, case; CR, current report; Multi, multicolored (including black); Ref, reference ^a^Tinea completely resolved after treatment. ^b^Tinea completely cleared after four weeks of treatment. ^c^Tinea lesions regressed after treatment. ^d^After treatment of tinea lesions, repeat potassium hydroxide preparation was negative for hyphae and fungal culture was negative for dermatophyte organism. ^e^Tinea resolved within three weeks. ^f^All tinea lesions cleared within weeks; however, lesions associated with allergic contact dermatitis to red tattoo ink became worse. ^g^Topical luliconazole made the lesions worse and was discontinued; all lesions cleared after 11 weeks of itraconazole. ^h^At the end of four weeks there was complete clinical cure of tinea lesions.

C	Location	Tattoo color	Lesion morphology	Topical treatment	Oral treatment	Ref
1	Left Forearm	Black	Asymptomatic red scaly plaque	Ketoconazole two percent cream for four weeks^a^	None	CR
2	Arm	Not stated	Pruritic circumscribed annular scaly plaque	Miconazole cream for four weeks^b^	None	[1] C2
3	Right Forearm, Left Thigh	Black	Pruritic, oozing, erosive annular patches and erythematous and infiltrating papules	Terbinafine cream^c^	Terbinafine 250 milligrams per day for one month^c^	[7]
4	Left Calf	Black and green	Circular red scaly plaque with raised red vesiculopustular edge and central resolution	Terbinafine one percent cream daily for 21 days^d^	Terbinafine 250 milligrams per day for 21 days^d^	[4]
5	Right Arm	Multi	Pruritic scaly annular plaques	Miconazole cream for three weeks^e^	Griseofulvin for three weeks^e^	[1] C1
6	Left Calf	Red	Pruritic red scaly patch	Ketoconazole two percent cream twice daily^f^	Terbinafine 250 milligrams per day^f^	[5]
7	Eyebrows	Black	Pruritic red patches	Luliconazole for two days; then white petrolatum^g^	Itraconazole: 50 milligrams per day for two days; then 100 milligrams per day for 11 weeks^g^	[3]
8	Leg	Black	Pruritic peripherally expanding concentric lesions with vesiculopustular border	Not stated	Not stated	[2]
9	Extremity	Black	Red plaque	Amorolfine cream daily for four weeks^h^	Itraconazole 100 milligrams twice daily for two weeks^h^	[6] C1
10	Extremity	Black	Red plaque	Amorolfine cream daily for four weeks^h^	Itraconazole 100 milligrams twice daily for two weeks^h^	[6] C2
11	Extremity	Black	Red plaque	Amorolfine cream daily for four weeks^h^	Itraconazole 100 milligrams twice daily for two weeks^h^	[6] C3
12	Extremity	Black	Red plaque	Amorolfine cream daily for four weeks^h^	Itraconazole 100 milligrams twice daily for two weeks^h^	[6] C4

Four men and four women with tattoo-associated tinea have been described; the sex was not provided for four patients. All the patients ranged in age from 18 years to 63 years (median, 29 years). The men ranged in age from 18 years to 52 years (median, 29 years) and the women ranged in age from 27 years to 63 years (median, 29 years).

The duration of the tattoo--from inoculation until the patient became aware of the dermatophyte lesion within the tattoo--varied from six days to nearly six years. Indeed, the tattoo duration prior to the discovery of tinea is likely to be influenced by the pathogenesis of tattoo-associated tinea in that individual. Six of the patients (with seven tattoos) developed tinea within one or less months after tattoo inoculation: six days to one month (median, two weeks). In contrast, six individuals experienced a later occurrence of dermatophyte infection ranging from more than two months (four patients) to either 45 months or 71.25 months after they received their tattoo.

The diagnosis of tinea was based only on the presence of hyphae observed on the potassium hydroxide preparation (one patient), demonstration of hyphae on the tissue specimen from a skin biopsy (one patient), or both observation of hyphae on the potassium hydroxide preparation and culture of the dermatophyte organism (ten patients). The identification of the dermatophyte included *Trichophyton rubrum* (four patients), *Epidermophyton floccosum* (two patients), *Microsporum canis* (two patients), *Microsporum gypseum* (one patient), and *Trichophyton tonsurans* (one patient). The organism was not cultured in two of the patients.

Tinea can be transmitted as an anthrophilic infection (from person to person), or a zoophilic infection (from animal to person) or a geophilic infection (from soil to person). A source for the tinea that developed in the tattoo was described for five of the patients and speculated--yet unconfirmed--for an additional individual; no source was provided for six of the patients. Autoinfection of tinea from another body area was suggested for two patients; the 35-year-old man had tinea pedis (potassium hydroxide preparation-positive and culture-positive for *Trichopyton rubrum*) and tinea cruris (potassium hydroxide preparation-positive) and the 52-year-old man had clinical findings of tinea pedis and toenail onychomycosis. One patient had an anthrophilic source of infection--her five-year old son. The woman with tattoo-associated *Trichophyton tonsurans *of her leg tattoo had a five-year-old son whose tinea capitis cultured the same dermatophyte organism; the researchers postulated that she had scratched her tattoo during the initial days after its inoculation with fingers that had contacted her son’s tinea-infected scalp.

Two women had a zoophilic source of their tattoo-associated tinea. The animal vector was either the pet cat or the pet dog. A 27-year-old woman developed a tattoo-associated *Microsporum canis *infection which appeared either six or 12 days after inoculation of black tattoos on her left thigh and right forearm, respectively; the veterinarian had evaluated the cat for hair loss which was attributed to ringworm and her tattoo-free sister had similar lesions on the abdomen. The second woman was 29-years old and had a tattoo-associated *Microsporum canis *infection which appeared 18 days after inoculation of a black-and-green-colored tattoo on her left calf; she had direct contact with her pet dog that had also been diagnosed with a culture-proven *Microsporum canis* mycosis.

One patient, a 63-year-old woman, developed tattoo-associated *Microsporum gysium* one month after cosmetic tattooing of her eyebrows. A geophilic source for the organism was favored since the dermatophyte is typically found in the soil. Since the patient had a pet chihuahua that she would sleep with each evening, the researchers postulated that the dog’s paws might have carried fungal organism contaminated soil into the bed which subsequently infected the healing tattoo. However, no skin lesions were found on the dog when it was examined by a veterinarian.

Tattoo-associated tinea has been described in 12 patients with 13 tattoos; a 27-year-old woman had two tattoos that each had a dermatophyte infection--one on the left thigh and one on the right forearm. Over 92 percent (12 of 13) of the dermatophyte-infected tattoos were on an extremity; these included either the upper extremity (with two on the arm and two on the forearm) or the lower extremity (with two on the calf and one on the thigh and one on the leg). The specific extremity was not specified for four patients. The last patient, a 63-year-old woman, had tinea that infected her eyebrows after cosmetic tattooing.

The color of the tattoo was described for 12 of the tattoos. Nine of the tattoos only had affected areas that were black. One tattoo was multicolored (including black) and the tinea-infected areas of one tattoo were both black and green. Only one patient did not have a tinea-infected portion of the tattoo that was black--the 52-year-old man with a red tattoo, that was infected by a dermatophyte, on his right calf.

The most common presenting feature was a new lesion or a progressively enlarging lesion within a tattoo. Six of the dermatophyte lesions were pruritic. The patient was either asymptomatic (one patient) or no symptoms were described (five patients) for the other tinea lesions.

The morphology of tattoo-associated tinea is variable. Tinea incognito refers to the non-classical presentation of superficial tinea infection which can occur in the setting of immune suppression when the patient is receiving either topical or systemic corticosteroids. Three patients presented with atypical appearing lesions secondary to tinea incognito; the lesions were pruritic erythematous patches in two patients--the 52-year-old man and the 63-year-old woman--and an asymptomatic red scaly plaque in the 18-year-old man.

Four patients had red plaques. Two patients had concentric, peripherally expanding plaques with central resolution and vesiculopustular borders. The other three patients had red, scaly or non-scaly, annular or non-annular, patches; one of the patients also had erosive and oozing lesions.

Treatment of tattoo-associated tinea--described in 11 of the patients--involved only topical agents (in two patients) or both topical and oral antifungal drugs (in nine patients). The topical antimycotic therapy included amorolfine cream (four patients), ketoconazole two percent cream (two patients), miconazole cream (two patients), terbinafine one percent cream (two patients), or luliconazole (one patient for only two days until she developed an allergic reaction to the agent). Oral antifungal drugs--used in nine of the patients--included itraconazole (five patients) terbinafine (three patients), or griseofulvin (one patient).

The response to therapy was described in 11 patients. There was complete resolution of the tattoo-associated tinea in all 11 individuals. Neither treatment nor follow-up was provided for the woman with leg tattoo-associated *Trichophyton tonsurans* whose son had tinea capitis.

The epidemiologic features and clinical characteristics of tattoo-associated systemic fungal infections are summarized in Table 4 and Table 5 [8-15]. Six patients with systemic fungal infection within a tattoo have been described. The individuals have been observed in the United States of America (two patients), Australia (two patients), the Czech Republic (one patient), and Finland (one patient).

**Table 4:** Epidemiologic features of tattoo-associated systemic fungal infections Abbreviations: A, age (years); Aus, Australia; Bx, biopsy; C, case; CR, Czech Republic; Cty, country; Cult, culture (fungal); Fin, Finland; KOH, potassium hydroxide preparation; Ic, immunocompetent; Im, immune status; Is, immunosuppressed; NS, not stated; PCR, polymerase chain reaction; Ref, reference; RNA, ribonucleic acid; Tat dur, Tattoo duration (the number of months from inoculation of the tattoo until the patient became aware of the dermatophyte lesion within the tattoo); USA, United States of America; <, less than; -, negative for; +, positive for ^a^This was performed on the teased tissue mount in 1.78 Molar (ten percent) potassium hydroxide stained with Parker ink. ^b^Fungal hyphae were identified on Grocott’s methenamine silver stain from each of the skin biopsies taken from the left forearm, right ankle, and left shin. ^c^Initial and repeat (two years later) biopsies and cultures were performed. Both biopsies were negative for the fungal organism; however, both fungal cultures were positive for the organism. In addition, the initial culture grew *Staphylococcus aureus*, group A *Streptococcus*, and a pigmented rapid-growing unclassified atypical *Mycobacterium* and the second culture grew *Staphylococcus aureus*, and *Enterobacter aerogenes*.

C	A	Sex	Im	Cty	Tat dur	Diagnosis	Source	Ref
1	24	Man	Ic	Fin	<1	Bx: - hyphae; Cult: Aspergillus fumigatus	Not known. The patient had a home-made tattoo performed by a non-professional tattooist friend. Researchers postulated contaminated instruments or ink, lack of aftercare, or building renovation-related fungal organism aerosolization at home or at work.	[10,11]
2	25	Man	Ic	Aus	69	Bx: + hyphae; KOH: + hyphae^a^; Cult: Saksenaea vasiformis	Not known. Acquisition of the forest soil organism is usally associated with severe trauma and/or immunosuppression; save for his tattoo, the patient was an otherwise healthy young man.	[12]
3	32	Man	Ic	USA	0.1	Bx: + organism; Cult: Sporothrix schenckii	Not known. Researchers postulated that not only the acquisition of the tattoo (inoculated by self-tattooing) may have been associated with non-sterile instruments or tattoo pigment but also that he exposed the tattoo to grass by mowing the lawn only wearing sandals on the day of tattoo inoculation.	[13]
4	33	Man	Is	USA	3	Bx: + hyphae^b^; 18s ribosomal RNA PCR: + organism; Cult: Purpureocillium lilacinum	Not known. He had end-stage renal disease secondary to polycystic kidneys; one year earlier, he had a kidney transplant. Seven months later, he had an acute cellular rejection that required treatment with plasmapheresis, rituximab, and bortezomib. Two months after his rejection, he received his tattoo. Researchers postulate that his immunosuppression or that the tattoo needle or ink was contaminated, or both caused his infection.	[14]
5	36	Man	Ic	Aus	3	Bx: - organism^c^; Cult: Sporothrix schenckii^c^	Not known. The patient experienced ritual Samoan body tattooing over the legs, buttocks, abdomen and back using tattooing combs made from a boar’s (male pig) tusk or bone and tattooing dye consisting of soot (collected either by scraping the inside of a tin drum that has been placed over a fire or from the burned inner surface of a coconut shell) and ink (made by crushing the seeds of a candle nut tree). Hence, his researchers postulated that either non-sterile instruments or pigment was used during the tattoo procedure.	[15]
6	NS	NS	NS	CR	NS	Cult: Acremonium species	Fungal cultures of contaminated tattoo ink (found in several batches from the United States) grew the fungal organism. Other suspected batches of tattoo ink were withdrawn from the French market during the summer of 2004 when they were analyzed and grew not only the fungal organism, but also Aeromonas species, Pseudomonas aeruginosa and Pseudomonas putida.	[8,9]

**Table 5:** Clinical characteristics of tattoo-associated systemic fungal infections Abbreviations: C, case; L, left; R, right; Ref, reference; Terb, terbinafine; Top Tx, topical treatment ^a^The systemic fungal infection resolved after four weeks; the patient had surgical revision of the necrotic tissue. ^b^He was clinically free of systemic fungal infection seven months after completion of treatment. ^c^The systemic fungal infection resolved after four months of itraconazole. ^d^The systemic fungal infection resolved after three months of voriconazole. ^e^Initially, his concurrent bacterial and mycobacterial infection was treated with minocycline. Subsequently, prior and during receiving itraconazole, his coexisting bacterial infections were concurrently treated with flucloxacillin 500 milligrams four times daily for four months and doxycycline 100 milligrams twice daily for four months. The bacterial infection resolved. At the completion of itraconazole therapy, there was full resolution, with no relapse, of the systemic fungal infection.

C	Location	Tattoo color	Lesion morphology	Top Tx	Oral treatment	Ref
1	Back	Black	Painful purpuric necrotic papules and pustules evolving into crusts.	Terb^a^	Voriconazole for four weeks.^a^	[10,11]
2	Left Forearm	Blue	Necrotic ulcer (ulcerated granulomatous lesion) and numerous small satellite lesions.	None	Potassium iodide 500 milligrams three times daily for 20 months; there was decreased compliance and recurrence. Then intravenous amphotericin: total dose of 1800 milligrams over 77 days.^b^	[12]
3	Dorsal Left Foot	Blue	Multiple firm, non-tender, erythematous papules and nodules.	None	Saturated solution of potassium iodide: five drops three times daily for three weeks and then ten drops three times daily for two weeks; stopped because of nausea. Then itraconazole 100 milligrams twice daily for four months.^c^	[13]
4	Left Forearm	Black	Asymptomatic five-millimeter dome-shaped pink papules.	None	Voriconazole for three months.^d^	[14]
5	Left Lower Thigh and Knee	Black	Nodules initially. Subsequently nodules and multiple small pyogenic abscesses.	None	Initially, saturated solution of potassium iodide for three months; stopped because of nausea and drowsiness. Two years later, itraconazole 100 milligrams twice daily for eight months.^e^	[15]
6	Arm	Not stated	Mycetoma	Not stated	Not stated.	[8,9]

Tattoo-associated Candida endophthalmitis has also been described in a 40-year-old immunosuppressed man (caused by a trauma-associated splenectomy during childhood). The development of his systemic fungal eye infection occurred within less than one week after he received a tattoo. However, in contrast to the other patients with tattoo-associated systemic fungal infections, his *Candida albicans* infection developed in his right eye instead of occurring within the tattoo [17].

The man had a black tattoo inoculated onto the deltoid area of his left proximal arm; three days later he developed a red right eye and reduced vision. One week after tattoo acquisition, he was evaluated by an ophthalmologist; the visual acuity of his right eye was 20/40 and the visual acuity of his left eye was 20/20. Additional evaluation of his right eye showed disc hyperemia; the anterior chamber had one plus cells and the vitreous body not only had two plus cells but also debris. Also, along the inferotemporal vascular arcade, a white fluffy chorio-retinal lesion was noted [17].

A diagnostic vitrectomy from his right eye was performed; the sample from the vitreous body grew *Candida albicans*. He was initially treated with intravenous amphotericin B (at a dose of two milligrams per kilogram) followed by daily oral fluconazole (at a dose of 200 milligrams per day). His visual acuity in both eyes was 20/20 and his right eye was normal at his four-month follow-up visit [17].

Five men with tattoo-associated systemic fungal infection within the tattoo have been described; the sex was not provided for the sixth patient. The patients ranged in age from 24 years to 36 years (median, 33 years). The immunologic status was provided for five of the six individuals: four patients were immunocompetent and one patient (the 33-year-old man) was immunosuppressed since he was a kidney transplant recipient.

The duration of the tattoo--from inoculation until the patient became aware of the systemic fungal lesion within the tattoo--varied from several days to 69 months (median, three months). Four of the patients (80 percent) noticed their systemic fungal infection lesion in three months (two patients) or less: either a couple of weeks or several days. Although the 36-year-old man was aware of this tattoo-associated fungal lesion after three months, he did not seek medical evaluation until four years later.

The diagnosis of tinea was based only on tissue biopsy for at least five of the patients. Microscopic examination of the tissue specimen demonstrated the fungal organism in three patients; however, biopsy-negative, culture-positive specimens were obtained in two patients. Fungal organisms were also observed on the potassium hydroxide preparation of a teased tissue mount in a 25-year-old man. In addition, 18s ribosomal ribonuclear protein (RNA) polymerase chain reaction confirmed the diagnosis of the fungal organism in the 33-year-old man.

The identification of the systemic fungal organism included *Sporothrix schenckii* (two patients), *Acremonium* species (one patient), *Aspergillus fumigatus* (one patient), *Purpureocillium lilacinum* (one patient), and *Saksenaea vasiformis* (one patient). The 36-year-old man with tattoo-associated *Sporothrix schenckii *also had concurrent bacterial infection of the nodular fungal lesions. In addition to the fungus, the initial cultures revealed *Staphylococcus aureus*, group A *Streptococcus *and an atypical (pigmented rapid growing) mycobacteria; repeat cultures also grew the fungus but also demonstrated *Staphylococcus aureus *and *Enterobacter aerugenes *[15].

Systemic fungal infections can be acquired by inhalation of spores. However, primary systemic fungal infections of the skin can be acquired percutaneously. Indeed, this is the proposed mechanism of pathogenesis for cutaneous infection in patients with tattoo-associated systemic fungal infections.

The infectious source of the tattoo-associated systemic fungal infection has only been definitively established for one of the individuals--contamination of the tattoo ink. Several batches of ink, that had been made in the United States, were discovered to be contaminated with *Acremonium* fungi. A patient, who had been reported in the Czech Republic, developed a mycetoma on the tattooed arm. Subsequently, additional investigation during the summer of 2004 revealed that several batches of tattoo ink were analyzed and withdrawn from the French market due to contamination by several germs including not only *Acremonium* species, but also bacteria of the genus *Aeromonas*, *Pseudomonas aeruginosa*, and *Pseudomonas putida *[9].

The infectious source of the tattoo-associated systemic fungal infection has been postulated to be the method of inoculation by either an amateur tattooist or by the patient themselves. The 24-year-old man with *Aspergillus fumigatus* within his back tattoo had a homemade tattoo performed by a non-professional tattooist; the person who inoculated his tattoo was a friend. An increased risk of adverse cutaneous events has been observed when tattoos are performed by amateurs, who are also referred to as backyard tattooists or scratchers. In addition, the investigators speculated several other possibilities for acquiring tattoo-associated *Aspergillus fumigatus*: contaminated instruments or tattoo ink, lack of appropriate aftercare, and/or aerosolization of fungal organisms from building renovation either at his home or at his work [10,11].

The second patient was the 32-year-old man with lymphocutaneous *Sporothrix schenckii *infection within his dorsal left foot tattoo. When the researchers discovered that he had performed self-tattooing, they considered the possibility that he may have used non-sterile instruments or tattoo pigment. In addition, the researchers hypothesized that he may have percutaneously acquired a fungal organism from the soil when he exposed the tattoo site to grass, on the same day after he inoculated his own tattoo, by mowing the lawn while wearing only sandals on his feet [13].

The investigators did not have a source of infection for the other three men with tattoo-associated systemic fungal infection [12,14,15]. They suggested contamination of the tattoo needle or tattoo ink that were used during the inoculation; however, this could not be proven. One of the men--the 33-year-old with the left forearm tattoo--had received a kidney transplant and had experienced an episode of acute cellular rejection that required aggressive treatment with immunosuppressive agents just two months before his tattoo was inoculated. Hence, he was immunocompromised and therefore susceptible to acquiring an opportunistic systemic fungal infection such as *Purpureocillium lilacinum *[14].

Over 93 percent (five of six) of the systemic fungal-infected tattoos were on an extremity. These included either the upper extremity (with two on the forearm and one on the arm) or the lower extremity (with two on the foot and one on the thigh). The last patient, a 24-year-old man, had *Aspergillus fumigatus *that infected his back a couple of weeks after tattooing.

The color of the tattoo was described for five of the tattoos. All the tattoos were dark colored. Three of the tattoos only affected areas that were black. Two of the tattoos were blue.

Two patients--the 32-year-old man with *Sporothrix schenckii *and the 33-year-old man with *Purpureocillium lilacinum*--had asymptomatic or non-tender skin lesions. The 24-year-old man with *Aspergillus fumigatus *had painful tattoo-associated systemic fungal skin lesions. The presence or absence of symptoms was not described in the other patients.

The morphology of tattoo-associated systemic fungal lesions was variable. Some of the lesions were pink or erythematous and firm. They appeared as individual (one patient) or multiple (five patients), intact (three patients) or ulcerated (two patients), papules (three patients) or nodules (two patients); in some of the individuals, multiple satellite lesions (one patient) or pustules (two patients) were also present. The lesion was only described as a mycetoma in one patient.

Treatment of tattoo-associated systemic fungal infections tinea--described in five of the patients--included both a topical agent and an oral antifungal drug (in one patient) or only oral therapy (in four patients). The 24-year-old man with *Aspergillus fumigatus *was successfully treated with topical antimycotic therapy (terbinafine) and one month of voriconazole. The 33-year-old man kidney transplant recipient with *Purpureocillium lilacinum *was also successfully treated with three months of voriconazole.

Three men were initially treated with potassium iodide. The two men with *Sporothrix schenckii *could not tolerate the medication because of drowsiness (one man) and/or nausea (both men); both were subsequently successfully treated with itraconazole. The third man was being treated with potassium iodide for 20 months but experienced a recurrence of *Saksenaea vasiformis *secondary to decreased compliance; his systemic fungal infection was subsequently successfully treated with intravenous amphotericin.

In summary, including this patient, tattoo-associated tinea corporis has been observed in 12 individuals with 13 tattoos. The tattoo-related superficial fungal infection most commonly occurred on an extremity in black tattoo ink. The dermatophyte infection usually appeared as a red, scaly or non-scaly, annular or non-annular, patch or plaque, with or without central resolution and vesiculopustular borders; however, prior corticosteroid treatment resulted in tinea incognito in three patients. Half of the patients developed tinea within one month or less after tattoo inoculation; the development of tattoo-associated dermatophyte during tattoo healing suggests several potential mechanisms of pathogenesis for the occurrence of the tinea infection: injury to the skin from the tattoo needle, or use of non-sterile instruments, or contaminated ink, and/or contact with a human or animal dermatophyte source. The other six patients developed the tinea infection more than two months after receiving their tattoo; the development of tattoo-associated tinea after tattoo healing raises the possibility of a tattoo ink-associated phenomenon such as the presence of nanoparticles, polycyclic aromatic hydrocarbons, or cytokine-enhancement, and/or immunocompromised cutaneous district creation. All patients had complete resolution of tattoo-associated tinea after treatment with topical and/or oral antifungal agents [1-7].

Tattoo-associated systemic fungal infection has been reported in six patients; however, several features were not described in one patient. The onset of infection after tattoo inoculation was either within less than one month (two men), three months (two men), or 69 months (one man). All the tattoos had dark ink: black (three men) or blue (two men). The lesions were non-painful (two men) or tender (one man). The lesions were described as papules or nodules that were either individual or multiple and intact or ulcerated; one man had a mycetoma. *Acremonium *species, *Aspergillus fumigatus*, *Purpureocillium lilacinum*, *Saksenaea vasiformis*, and *Sporothrix schenckii *were the diagnosed systemic fungal organisms. The cause of the systemic fungal infection was only confirmed for one patient: contaminated ink. Non-professional tattoo inoculation, infected tattooing tool and/or ink in an immunosuppression host, and contaminated ritual tattooing instruments (boar tusk or bone) and dye (soot and crushed candlenut tree seeds) were postulated as the source of the infection in some of the other men. Systemic antifungal drug therapy resulted in complete resolution of the tattoo-associated systemic fungal infection [8-15].

It remains to be determined whether the relationship between tattoos and the development of either dermatophyte infection or systemic fungal infection is causative or coincidental. The increasing number of reported individuals with tumor-associated dermatophyte infections introduces the possibility that the tattoo may elicit this cutaneous response. This could result from a direct or an indirect effect of tattooing [1-7]. 

Tattoo-associated tinea has been observed in two clinical settings based on the duration of time between the inoculation of the tattoo and the appearance of the dermatophyte infection within the tattoo: early tinea infection on a recent (healing) tattoo--reflecting a direct effect of the tattooing process on the development of the dermatophyte infection--in six patients or late dermatophyte infection on an older (and healed) tattoo--reflecting an indirect effect of the tattooing process--in six patients. Tattoo needle-related injury to the skin, or instruments that were not sterile, or ink that was contaminated, and/or contact with a human or animal dermatophyte source may have promoted the development of tattoo-associated tinea in healing tattoos [1-4,7]. In contrast, reactions caused by the tattoo ink, including not only ink-associated nanoparticles, polycyclic aromatic hydrocarbons, and cytokine-related phenomenon but also the creation of an immunocompromised cutaneous district from diminished cellular and humoral immunity, may have prompted the pathogenesis of tinea occurring in healed tattoos [1,5,6,18].

The paucity of tattoo-associated systemic fungal infections raises the possibility that the infection that occurred within the tattoo of some of the patients may have merely been a coincidence. However, the tattoo duration was less than one month prior to the onset of the associated systemic fungal infection for two of the men. Both received non-professional tattoos (that were either home-made and performed by a friend or self-inoculated) and may have had exposure to the fungal organisms--either *Aspergillus fumigatus *or *Sporothrix schenckii*--during or shortly after the creation of their tattoo and with the time associated with the healing process [10,11,13].

Also, although the systemic fungal infection was initially noticed three months after tattooing for two of the men, their investigators favored--but could not document--exposure to the organisms during tattooing. The patients included the man who experienced ritual Samoan body tattooing and developed sporotrichosis and the immunocompromised kidney transplant recipient who acquired a *Purpureocillium lilacinum *infection. Hence, it might be speculated that both men acquired the systemic fungal infection during either the inoculation or healing of their tattoo but experienced a delay in the development of the clinical manifestations; indeed, an immunocompromised district may have been created associated with the tattooing process or the tattoo ink or both that allowed the systemic fungal infection to eventually manifest [14,15].

The researchers were unable to provide a mechanism for pathogenesis for the *Saksenaea vasiformis *that appeared six years and nine months after tattooing in the otherwise healthy 25-year-old man. Human zygomycosis rarely has been observed in association with this fungal organism; indeed, the affected individual is typically either immunosuppressed or has experienced severe trauma. Hence, in this patient, the tattoo-associated systemic fungal infection may have merely been coincidental [12].

The patient with an *Acremonium *species-related mycetoma on his arm tattoo developed the systemic fungal infection secondary to contaminated tattoo ink [8,9]. An older study evaluating commercial tattoo inks used in daily practice in a tattoo parlor in Europe observed that all the samples were sterile and cultures for pyogenic bacteria, mycobacteria, and fungus were all negative for these infectious organisms [19]. However, a more recent investigation of 83 unopened commercial tattoo and permanent makeup inks, purchased from 13 companies and available in the United States, for infectious pathogens demonstrated contamination by either bacteria (33 inks), fungi (two inks), or both bacteria and fungi (seven inks) [20]. Hence, inoculation of contaminated tattoo ink is a potential cause for the development of not only tattoo-associated tinea infections but also tattoo-associated systemic fungal infections.

## Conclusions

Tattoo-associated infections include not only bacteria, mycobacteria, and viruses, but also fungal infections. The features of a man who developed tattoo-associated dermatophyte infection in the black inked portion of a tattoo on his left arm less than one-month after its inoculation was described. The relationship between tattoos and the development of either dermatophyte infection or systemic fungal infection remains to be determined. In patients with tumor-associated tinea, several researchers favor that the tattoo inoculation can be implicated as a causative factor in the development of the subsequent dermatophyte infection. However, in some of the men with a tattoo-associated systemic fungal infection, the occurrence of the systemic fungal infection within the tattoo may have merely been coincidental.
